# Duties of the Medical Profession

**Published:** 1868-05-01

**Authors:** 


### *Duties of the Medical Profession.*

Valedictory Address to the last graduating class of the Medical Department of the University of Michigan, by E. O. Haven, D.D., LL.D., President of the University. An eloquent and high-toned tribute to the profession, which alone would stamp its author as a clear-headed thinker and an acute observer, free from *isms* and cant of all sorts. We have marked several passages for publication in this journal, as soon as space will permit. The bold stand taken by President Haven against accepting the *bribe*, although unsuccessful in result, entitles him to the thanks of the profession, as his high attainments and purity of character demand the esteem and confidence of the community.

